# Multimodality Imaging Approach to Infective Endocarditis: Current Opinion in Patients with Congenital Heart Disease

**DOI:** 10.3390/jcm14061862

**Published:** 2025-03-10

**Authors:** Nunzia Borrelli, Jolanda Sabatino, Alessia Gimelli, Martina Avesani, Valeria Pergola, Isabella Leo, Sara Moscatelli, Massimiliana Abbate, Raffaella Motta, Rosalba De Sarro, Jessica Ielapi, Federico Sicilia, Marco Alfonso Perrone, Pier Paolo Bassareo, Berardo Sarubbi, Giovanni Di Salvo

**Affiliations:** 1 Adult Congenital Heart Disease and Familiar Arrhythmias Unit, Monaldi Hospital, 80131 Naples, Italy; 2 Department of Experimental and Clinical Medicine, Magna Graecia University Catanzaro, 88100 Catanzaro, Italy; 3 Fondazione CNR, Regione Toscana “Gabriele Monasterio”, 56124 Pisa, Italy; 4 Pediatric Cardiology Unit, Department of Women’s and Children’s Health, University of Padua, 35128 Padua, Italy; 5 Dipartimento di Scienze Cardio-Toraco-Vascolari e Sanità Pubblica, University Hospital of Padua, 35128 Padua, Italy; 6 CMR Department Royal Brompton and Harefield Hospitals, Guy’s and St Thomas’ NHS Foundation Trust, London SW3 6PY, UK; 7 Inherited Cardiovascular Diseases, Great Ormond Street Hospital, Children NHS Foundation Trust, London WC1N 3JH, UK; 8 Institute of Cardiovascular Sciences, University College London, London WC1E 6BT, UK; 9 Clinical Pathways and Epidemiology Unit, Bambino Gesù Children’s Hospital IRCCS, 00165 Rome, Italy; 10 Division of Cardiology and Cardio Lab, Department of Clinical Science and Translational Medicine, University of Rome Tor Vergata, 00133 Rome, Italy; 11 School of Medicine, University College of Dublin, Mater Misericordiae University Hospital, D07 R2WY Dublin, Ireland

**Keywords:** congenital heart disease, infective endocarditis, multimodality imaging, personalized medicine

## Abstract

Although advances in medical and surgical management have significantly improved clinical outcomes, infective endocarditis (IE) remains a significant threat to patients with congenital heart disease (CHD). The complexity of cardiac anatomy, the presence of prosthetic materials, and the emergence of novel pathogens pose unique diagnostic challenges in this specific population. However, the use of personalized imaging, integrating the strengths of each modality, has the potential to refine the diagnostic process, thereby optimizing diagnostic accuracy, guiding therapeutic decisions, and, ultimately, improving patient clinical outcomes. This review delves into the critical role of the multimodality imaging approach in the care of patients with IE and CHD, underscoring the importance of tailored and patient-centered management strategies in this vulnerable cohort.

## 1. Introduction

Patients with congenital heart disease (CHD) are at an increased risk of developing infective endocarditis (IE), which often leads to significant morbidity and increased mortality rates [1].

While hemodynamic alterations and frequent use of prosthetic materials contribute indeed to an increased susceptibility to IE, recent observations indicate a remarkable shift in epidemiology. Historically, the disease was largely confined to patients with unoperated CHD, cyanotic heart disease, or rheumatic heart disease, with Streptococcus species most implicated as the etiologic organisms. In contrast, in the modern era, IE most often occurs in patients with repaired CHD, including those with prosthetic valves or other implanted materials. In addition, there has been an increasing predominance of Staphylococcus species as the etiologic organisms [1,2].

However, diagnosis of IE in patients with CHD is particularly challenging due to complex anatomy, prosthetic materials, and frequent right-sided involvement, necessitating advanced imaging and high clinical suspicion. The current guidelines recommend a multimodality imaging approach to diagnosing patients with suspected IE. Evidence of valve or intracardiac material involvement by various imaging techniques is considered a major diagnostic criterion. Although echocardiography represents the first-line technique, Cardiac Computed Tomography (CCT), Cardiac Magnetic Resonance (CMR), and nuclear imaging may play an important role by providing important information that may confirm the diagnosis. These imaging techniques may indeed depict cardiac involvement, as well as the presence of IE-related peripheral complications [3].

In this review we will provide a comprehensive overview on the current role of multimodality imaging in the evaluation of IE in patients with CHD, highlighting the advantages and limitations of the different imaging modalities and exploring their clinical implications.

## 2. Left-Sided Infective Endocarditis (LSIE) in Congenital Heart Disease

### 2.1. Introduction to LSIE

In patients with CHD, the left side of the heart is the most common site for infection, a trend that aligns with observations in the general population [4]. IE is particularly prevalent among patients with complex heart lesions, as well as those with simple lesions such as congenital valve anomalies, such as bicuspid aortic valve (BAV) and mitral valve abnormalities, and prosthetic valves [5,6,7,8]. A smaller number of studies on infective endocarditis in the pediatric population confirm the same trend also in children [1,9].

BAV is among the primary contributors to left-sided infective endocarditis (LSIE) [8,10]. Studies have shown that individuals who have undergone aortic valve replacement face a significantly higher incidence of IE compared to those with unoperated valves [11]. Mitral valve prolapse (MVP) is another common congenital anomaly, with an estimated prevalence of 1–3%. This is defined as a myxomatous degeneration and superior displacement (>2 mm) of the mitral valve (MV) leaflet(s) with various degrees of associated regurgitation. LSIE are particularly prevalent in patients with MVP presenting with moderate to severe mitral regurgitation or a flail mitral leaflet [12,13,14].

According to the latest clinical guidelines, both BAV and MVP are categorized as intermediate-risk conditions for IE [3]. However, emerging research suggests that patients with BAV and MVP experience a higher frequency of IE, commonly caused by viridans group streptococci and odontogenic infections [15,16]. These findings indicate a clinical profile similar to that of patients classified as high-risk for IE, warranting consideration for reclassification of BAV and MVP as high-risk conditions necessitating antibiotic prophylaxis [17].

Moreover, patients with prosthetic heart valves, whether aortic or mitral, are at heightened risk for developing LSIE. In a wide national-based cohort study, compared to those with mechanical valves, individuals with biological valves have presented a greater risk of adverse outcomes, including IE, as well as higher mortality and an increased likelihood of requiring subsequent valve surgery [18].

### 2.2. Role of Echocardiography

Since the first echocardiographic description using M-mode in 1973 [19], echocardiography has taken on an increasingly central role in the diagnosis, management, and prognosis assessment of patients with IE [20]. A decisive contribution was made by the development of two-dimensional, three-dimensional, and transesophageal echocardiography (TEE), which has significantly improved the non-invasive detection of vegetations, with an 80% sensitivity for transthoracic mode and a 95% sensitivity for transesophageal studies [21].

**Figure 1:**
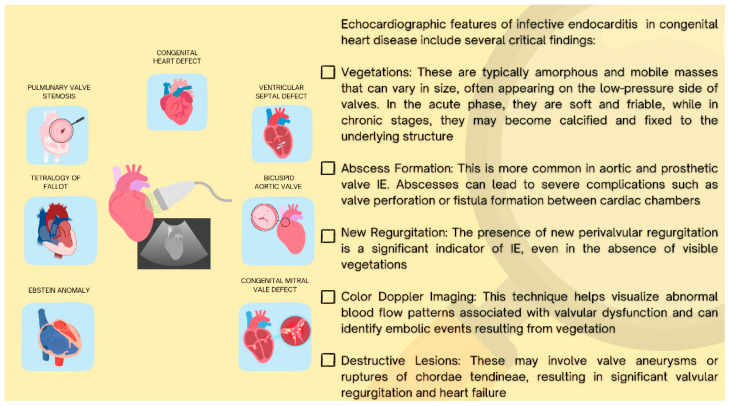
Echocardiographic major findings in infective endocarditis associated with congenital heart disease.

The main echocardiographic criteria (Figure 1) for the diagnosis of infective endocarditis include the presence of vegetations, perivalvular complications (such as abscesses, pseudoaneurysms, and prosthetic valve dehiscence), intracardiac fistulas, and valve perforations or aneurysms, which are characteristically similar in both left and right-sided IE [3]. In addition, color Doppler assessment of the hemodynamic implications of any valvular abnormalities is essential [22].

**Figure 2:**
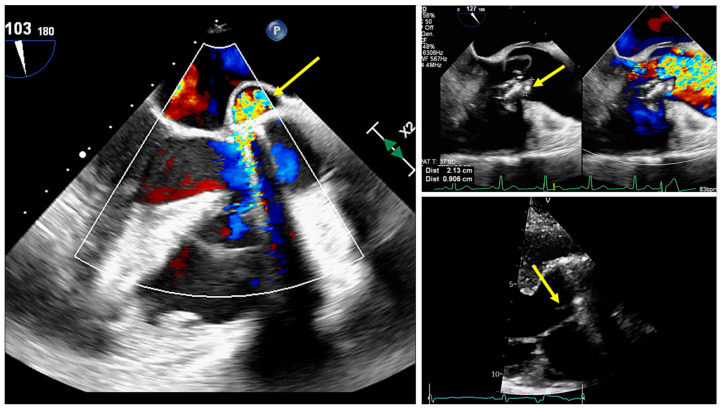
In clockwise order: transesophageal echocardiographic image of a pseudoaneurysm of mitral-aortic intervalvular fibrosa in a patient with prosthetic aortic valve and coarctation of aorta; transesophageal echocardiographic image of vegetation attached to a bicuspid aortic valve; transthoracic echocardiographic image of vegetation on prosthetic aortic valve. Yellow arrows point to pseudoaneurysm (image on the **left**) and to vegetations (images on the **right**).

Although the modified Duke criteria are universally applicable for diagnosing suspected IE, specific factors unique to patients with CHD must be considered. For instance, transthoracic echocardiography often proves suboptimal in adult patients with CHD due to their history of multiple surgical interventions or to the presence of conduits, prosthetic valves, shunts, and abnormal anatomical connections [23]. Consequently, TEE should be considered a priority diagnostic tool in most adult patients with CHD [24] (Figure 2). TEE is recommended due to its superior spatial resolution, which allows for more precise visualization of vegetation, particularly in patients with complex CHD, those with prosthetic valves, or those with Staphylococcus aureus infections [25,26,27].

However, detecting infections involving stents or other prosthetic materials—frequently affecting branch pulmonary arteries, pacing wires, or conduits—can remain challenging, even with TEE. Recently, three-dimensional (3D) echocardiography has also offered added value compared to two-dimensional (2D) TEE, providing more detailed information for excluding IE and for better surgical planning, thanks to the ability to accurately measure the size of vegetations and other anomalies [21,28] (Supplementary S1). However, its lower temporal and spatial resolution could be a limitation in identifying very small and highly mobile lesions [27].

It is crucial to emphasize that even with negative TEE findings in patients with prosthetic valves and positive blood cultures, a high index of suspicion for IE is warranted. These patients are at high risk for adverse outcomes and require prompt initiation of appropriate antimicrobial therapy. This approach is supported by recent evidence [29], which highlights the importance of clinical judgment and early treatment initiation in these challenging cases.

### 2.3. Role of Cardiac Computed Tomography

In patients with CHD, LSIE poses unique diagnostic and management challenges due to complex anatomical substrates, the presence of prosthetic material, and previous surgical interventions. CCT has emerged as a powerful adjunct to echocardiography in evaluating LSIE in this population. LSIE in CHD frequently involves the detection of vegetation, with periannular abscesses and pseudoaneurysms being common complications. While TTE and TEE are the primary imaging modalities, their diagnostic utility can be limited in CHD patients. Artifacts from prosthetic valves, anatomical alterations, and suboptimal acoustic windows often obscure key findings, necessitating additional imaging modalities [30,31].

CCT offers distinct advantages in such scenarios. Its high spatial resolution enables detailed visualization of vegetations, valve leaflet thickening, and calcifications, even in the presence of prosthetic material. Additionally, three-dimensional reconstruction allows for an accurate assessment of the perianular region, where abscesses and pseudoaneurysms are commonly found. Studies have demonstrated that CCT provides superior sensitivity in detecting paravalvular complications compared to echocardiography, particularly in prosthetic valve endocarditis [30,31].

In a recent meta-analysis, CCT was shown to reliably identify complications of infective endocarditis, including leaflet perforations, abscesses, and pseudoaneurysms, with diagnostic accuracy comparable to TEE [32]. This capability is particularly relevant in CHD patients, where anatomical distortions and surgical repairs often complicate traditional imaging approaches.

Moreover, systemic embolization is a frequent and severe complication of LSIE, with embolic events often involving the brain, spleen, or kidneys. CCT provides a comprehensive evaluation of embolic phenomena, enabling the detection of systemic emboli and infarcts [33]. In CHD patients, where the risk of embolization is further increased by turbulent blood flow across abnormal anatomical structures or prosthetic valves, CCT’s ability to detect distal embolic events significantly enhances clinical decision-making. Nagiub et al. highlighted the value of CCT in detecting embolic phenomena in young patients with CHD and LSIE, demonstrating its capability to identify splenic infarcts and embolic strokes that were missed by other imaging modalities [30].

**Figure 3:**
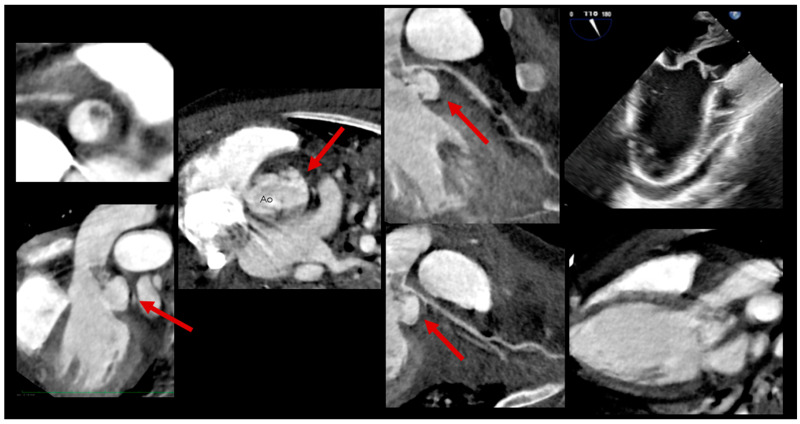
Infective aneurysm of the aortic valve with leaflet destruction, due to endocarditis on a bicuspid aortic valve (on ECMO); subaortic aneurysm with systolic compression of the LMCA and fistulization to the left atrium (LA); abscess extension to the mitral annulus with abscess involvement of the left atrial myocardium and fistulization to the left atrium. Red arrows point to infective aneurysms.

**Figure 4:**
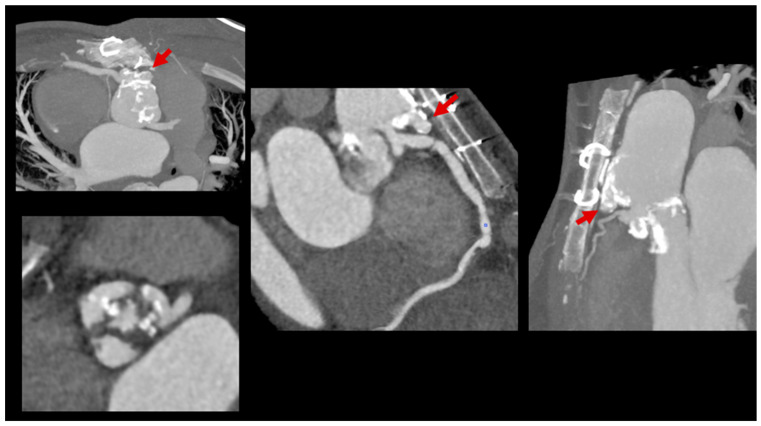
Severe subvalvular aortic stenosis post-surgery, followed by endocarditis, now dysplastic and calcified aortic valve, with pseudoaneurysms in the anterior wall of the ascending aorta, adjacent to the sternum and ligaments, near the origin of the right coronary artery. Red arrows point to pseudoaneurysm.

The incorporation of CCT into diagnostic pathways for LSIE is increasingly supported by evidence from the congenital heart population (Figure 3 and Figure 4). By complementing echocardiography, CCT has become instrumental in the detection of complications that directly influence therapeutic decisions, such as the timing of surgical intervention. The 2023 Duke criteria now recognize the role of CCT in providing major diagnostic criteria for infective endocarditis, further cementing its importance in clinical practice [34].

### 2.4. Role of Cardiac Magnetic Resonance

Despite its key role in the evaluation of patients with CHDs, CMR has limited usefulness in the diagnostic and prognostic assessment of patients affected by IE. The modality has, in fact, a lower temporal resolution compared to echocardiography, which may represent a limitation when dealing with small and highly mobile vegetation [35]. Nevertheless, an advanced multimodality imaging approach, including CMR, is recommended by the most recent guidelines in case of diagnostic uncertainty to improve diagnostic accuracy and assess related complications [3]. In addition, CMR can be useful when tissue characterization is needed, as in the case of suspected mass or concomitant myocardial or pericardial disease [3].

BAV is one of the most common left-sided CHDs, reported in about 0.5–2% of the general population [36,37]. Individuals with BAV have not only an increased risk of valve-related complications, such as stenosis or regurgitation, but also a greater risk of developing IE [3]. The heavy valve calcifications associated with the condition may represent a limitation in TTE image interpretation. In this context, CMR represents an ionizing radiation-free alternative to CT to assess both valve function and aortic diameters in case of associated aortic dilatation/coarctation [38]. A stack of cine images can indeed be acquired through the aortic valve to assess planimetric aortic valve area. Phase contrast (PC) images acquired at aortic valve level also allow measurement of aortic peak velocity and degree of aortic regurgitation. Attention should be paid to setting an appropriate velocity encoding sensitivity (VEnC) in case of aortic stenosis to minimize the occurrence of aliasing [39]. These sequences may be particularly useful in case of eccentric jets of aortic regurgitation secondary to cusps perforation or destruction, not always easy to accurately quantify with transthoracic echocardiography (TTE) [40].

The higher spatial resolution of CMR can also be useful to distinguish aortic valve stenosis from subaortic stenosis, where the left ventricular outflow tract is narrowed by a fibrous ring, a muscular ridge, or a fibromuscular tunnel, either alone or in various combinations [35]. A secondary, dynamic obstruction can also be seen in patients with hypertrophic cardiomyopathy and is associated with a higher incidence of IE [3]. CMR allows quantification of mass in the left ventricle, a more accurate assessment of the right ventricle or apical involvement in case of hypertrophic cardiomyopathy, and visualization and quantification of myocardial fibrosis for better prognostic assessment [41].

Recent studies showed that almost 20% of all IE cases are related to prosthetic valve implantation [42]. In the presence of a perivalvular abscess, CMR shows soft tissue thickening around the aortic root with hyperenhancement at late gadolinium enhancement surrounding an area with no signal (abscess cavity) [43,44]. Pseudoaneurysms are another rare but possible complication of prosthetic valve endocarditis. These are wall ruptures resulting in the formation of a sac-like structure contained by surrounding tissue (either pericardium, tissue adherence, or clotting products), usually presenting with a narrow neck. In contrast, true aneurysms have a broad base and are contained by all the myocardial wall layers [45]. An untreated infection may also result in abnormal communication between two cavities [3]; the resulting intracardiac shunt can be properly visualized and quantified by Qp/Qs assessment using PC sequences acquired at the shunt level (for visualization) and at pulmonary and aortic levels (for quantification) [46].

Parachute mitral valve is a rare congenital anomaly wherein all chordae tendineae (shortened and fibrotic) converge on a single papillary muscle, restricting the excursion of the MV leaflets, leading to valve stenosis in most cases [47]. Parachute-like asymmetric MV is a more common anomaly than true parachute mitral valve, characterized by the presence of two papillary muscles: one is morphologically normal, while the other is elongated with few or no chordae tendineae, leading to an eccentric MV orifice [48]. Case reports have indicated an increased susceptibility to IE in these patients, likely due to the turbulent blood flow across the abnormal valve [47]. These two conditions may be challenging to distinguish; CMR cine images can, in this case, accurately assess the number and attachment of MV papillary muscles [48]. MVP is another common congenital anomaly, associated with an increased risk of IE. Although accurately detected by echocardiography, CMR can be useful in unveiling associated mitral annulus disjunction and myocardial fibrosis as areas of late gadolinium enhancement (LGE), a predictor of arrhythmic sudden cardiac death in this population [49].

## 3. Right-Sided Infective Endocarditis (RSIE) in Congenital Heart Disease

### 3.1. Introduction to RSIE

RSIE is relatively rare in the general population, occurring in about 5–10% of endocarditis cases and involving mostly the tricuspid valve (TV). By contrast, it is significantly more common among patients with complex CHD, in whom the pulmonary valve is particularly, although not exclusively, affected [3]. Indeed, patients after tetralogy of Fallot (ToF) repair, placement of right ventricle-pulmonary artery conduit, with residual pulmonary valve (PV) stenosis or regurgitation, and a history of percutaneous/surgical valve replacement, are at heightened risk [50,51].

Several studies have also demonstrated that pulmonary valve replacement significantly elevates the risk of developing RSIE [52], with the transcatheter PV procedure carrying a higher risk compared to surgical pulmonary valve replacement [53]. Research shows that the cumulative incidence of RSIE following transcatheter PV procedure ranges from 5.14% to 11.8% [54,55,56]. Among valve types, the Melody valve has the highest IE incidence compared to the Sapien valve [57]. Data on the newer P-Venus valve are limited to a few cases [5,58], requiring longer follow-up for conclusions. Moreover, unrepaired ventricular septal defects (VSDs) with left-to-right shunts elevate 11–15-fold IE risk compared to the general population, with both the pulmonary and tricuspid valves commonly affected [59,60,61]. Finally, patients with Ebstein’s anomaly also present a heightened risk of developing IE, especially in the presence of additional risk factors such as heroin abuse [62] or cardiac devices [63]. The main intracardiac complications are the development of severe valvular insufficiency or stenosis, which often requires surgical intervention, while the primary extracardiac complication of RSIE is septic pulmonary embolization, which can result in significant respiratory compromise and further systemic complications [61,64].

### 3.2. Role of Echocardiography

TTE is the first-line imaging method for identifying RSIE in CHDs. Right-sided heart structures are positioned anteriorly in the chest and are closer to the TTE transducer; thus, the acoustic window should be adequate for an anatomic study, especially in young patients [20]. However, multiple surgeries, scars, conduit placements in extra-anatomic positions, stents or metallic devices, and high thoracic impedance make multiple and off-axis views necessary to investigate right-sided structures when IE is suspected [3].

**Figure 5:**
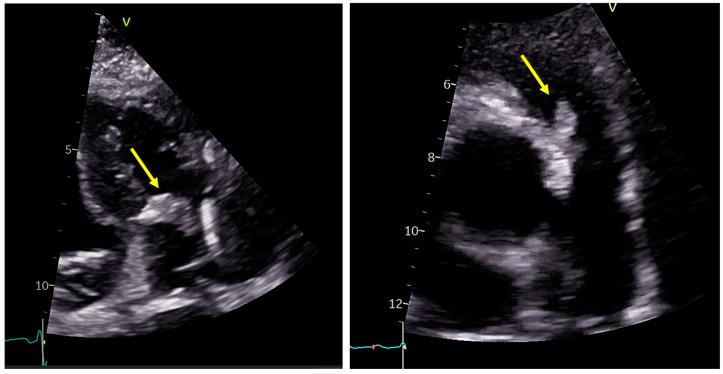
Transthoracic echocardiographic images of vegetations on the tricuspid valve (on the **left**) and pulmonary conduit (on the **right**). Yellow arrows point to vegetations.

In most patients, tricuspid valve, right ventricular outflow tract (RVOT), and pulmonary valve function can be adequately assessed with color and continuous wave (CW) Doppler. By contrast, vegetation may be challenging to visualize with TTE, especially on the PV and in patients with the features mentioned above [3,20] (Figure 5). In this context, a sudden rise in transvalvular gradient observed during follow-up imaging may represent an indirect sign of possible IE and suggest more advanced imaging, such as TEE [64].

TEE can often identify vegetation, which is usually located on the atrial side of the TV and the ventricular side of the PV. Additionally, TEE enables the evaluation of valvular function and the detection of abscesses, which can arise as a complication of LSIE. TEE is also indicated in patients with cardiac implantable electronic devices because TTE sensitivity for detecting vegetation attached to pacemaker leads is limited and in those with negative/inconclusive TTE [20,65,66]. Lastly, it might be helpful to identify uncommon infection localizations, such as the Eustachian valve or the Chiari network [67]. However, it should be remembered that identifying vegetation on the PV can be difficult even with TEE, particularly in patients with a prosthetic valve in the pulmonary position [68]. In this context, biplane imaging and live 3D TEE in expert hands might improve the diagnosis. Notably, three-dimensional echocardiography is no longer limited to adult patients, thanks to the development of pediatric transesophageal probes that can be used in infants weighing at least 5 kg [69].

TEE views for RSIE include [70]:

- Upper esophageal at 0°–10°: to investigate the main pulmonary artery and pulmonary branches;
- Mid-esophageal at 0°–10° and 50°–70°: to assess the right atrium, TV, subpulmonary region, and PV;
- Transgastric at 50°–90°: this view allows for an assessment of any residual obstruction in the RVOT, as well as valvular and supravalvular areas, because the ultrasound beam often aligns with the blood flow direction.

Intracardiac echocardiography might also be useful in selected cases, especially in patients with suspected prosthetic PV, cardiac device-related endocarditis, or in patients who do not tolerate TEE or in whom TEE is not feasible [71].

### 3.3. Role of Cardiac Computed Tomography

CCT has emerged as a pivotal tool in the diagnostic work-up of RSIE in patients with CHD. Traditional imaging techniques such as TTE and TEE often face limitations in visualizing vegetation and complications in RSIE, especially in the presence of prosthetic materials or complex anatomical substrates. CCT provides high-resolution imaging and 3D reconstruction capabilities, offering anatomical details that complement echocardiographic findings [30,31,72].

TTE, though widely used, is limited by its lower sensitivity for detecting right-sided vegetations, particularly in non-intravenous drug users [72]. TEE may not adequately image anteriorly positioned structures, such as the pulmonary valve and right ventricle-pulmonary artery conduit, due to acoustic shadowing and artifacts from prosthetic material [31].

**Figure 6:**
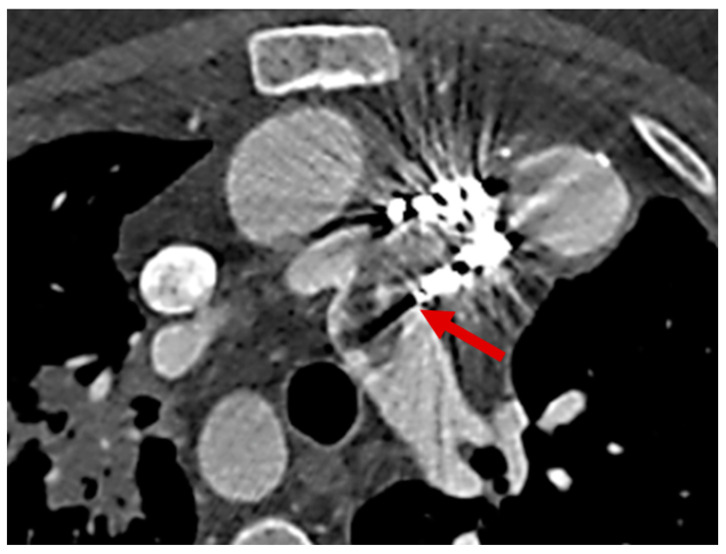
Septic emboli between pulmonary artery and pulmonary branches in a patient with tetralogy of Fallot and pulmonary valve replacement. Red arrow points to septic emboli.

CCT has proven highly effective in overcoming these challenges. Its ability to delineate vegetations, assess valvular thickening, and identify associated complications, such as pseudoaneurysms and abscesses, has made it a valuable adjunct to echocardiography. For example, studies have demonstrated that CCT reliably detects vegetation on prosthetic pulmonary valves and conduit walls, even in cases where echocardiographic imaging is inconclusive [30] (Figure 6).

Moreover, pulmonary complications, including septic emboli and infarcts, are hallmark features of RSIE and can significantly impact patient outcomes. CCT is uniquely suited to evaluate these complications, as it provides detailed visualization of the pulmonary vasculature and lung parenchyma. In a study by Thaler et al., CCT was instrumental in diagnosing pulmonary septic emboli and infarcts in CHD patients with suspected RSIE, findings that were critical for guiding clinical management [33].

The integration of CCT into diagnostic algorithms for RSIE is supported by its inclusion in updated Duke criteria. CCT’s ability to identify vegetations, abscesses, and pseudoaneurysms with high specificity and sensitivity enhances its utility in confirming endocarditis in complex cases [34]. Additionally, CCT can evaluate prosthetic valve function and structural integrity, providing a comprehensive assessment that informs surgical decision-making.

CCT also offers the unique advantage of evaluating the anatomical relationships between the coronary arteries, cardiac structures, and the sternum. This capability is particularly valuable in CHD patients with a history of multiple surgical interventions. Preoperative planning often requires detailed knowledge of the proximity of coronary arteries and other critical structures to previous surgical sites to minimize risks during re-sternotomy [30].

The role of CCT in RSIE extends beyond diagnosis to include preoperative planning and the evaluation of treatment efficacy. For patients with prosthetic material or CHD, where echocardiography alone may be insufficient, CCT serves as a non-invasive alternative with high diagnostic yield. Furthermore, advancements in radiation dose reduction and image reconstruction techniques have expanded the applicability of CCT in pediatric and young adult populations.

### 3.4. Role of Cardiac Magnetic Resonance

CMR provides detailed information about cardiac anatomy and function, blood flow patterns, and myocardial tissue characterization without the use of ionizing radiation and overcoming the limitation of poor acoustic windows [73,74]. In the context of IE in CHD patients, the role of CMR is somewhat limited, due to its lower spatial resolution compared to CCT and lower temporal resolution relative to echocardiography [3,6,75], and the frequent presence of prosthetic valves or other metallic devices [3,76]. Additionally, its relatively long acquisition times and the need for patient cooperation make it more complex to perform, particularly in the pediatric population [77].

However, CMR can be valuable in cases of diagnostic uncertainty or when additional information is needed [75,76,78]. The cardiac structural and functional evaluation is achieved through the application of 2D and 3D steady-state free precession (SSFP) cine imaging [74,79]. These sequences also enable the assessment of valvular damage and related complications, including abscess or pseudoaneurysm formation [3,26]. Advanced techniques like 3D and four-dimensional (4D) flow imaging, as well as 3D whole-heart images, allow for a comprehensive assessment of vascular and valvular blood flow and hemodynamic status, ideally without the use of contrast agents to avoid potential infection dissemination [80,81].

Tissue characterization is possible with the application of T1- and T2-weighted imaging, which provides insights into myocardial edema, fat infiltration, and scar content [74,79]. Additionally, the application of LGE permits the detection and quantification of myocardial fibrosis or scarring, which may suggest the presence of associated myocarditis, while delayed hyperenhancement supports the diagnosis of pericarditis [74,79,82].

Finally, the non-invasive nature and radiation-free profile make CMR an ideal option for CHD patients of all ages, from the pediatric period to adulthood, assisting in the management, including in cases of endocarditis [73,74,83].

The evaluation of RSIE in patients with CHD presents significant diagnostic challenges, primarily due to the visualization of the involved structures using standard echocardiography views [3]. Although echocardiography remains the first-line imaging modality for evaluating pulmonary valve and RVOT, a multimodal approach is often required, integrating CMR and CCT to obtain a more accurate anatomical and functional assessment [4,84].

CMR plays a complementary role in case of uncertainty to differentiate right atrial structures that may be associated with IE, such as the Chiari network or the Eustachian and Thebesian valves, and congenital defects like Ebstein’s anomaly of the tricuspid valve or cor triatriatum dexter [61,85,86,87]. A multimodal imaging approach is essential in assessing IE in patients with complex right-sided CHD, such as those with ToF or double outlet right ventricle (DORV) [75,88,89,90]. In these cases, CMR provides detailed pre- and post-operative evaluations, offering information on morphology, function, and hemodynamic status, as well as revealing anomalies underlying the infectious disease, such as pulmonary stenosis and regurgitation, right ventricle dilatation, and residual ventricular septal defects [91,92,93]. The utility of CMR also extends to the evaluation of infected right-sided distal structures, such as stents or other prosthetic material in the branch pulmonary arteries [26]. However, use of CMR is limited if the endocarditis involves metallic prosthetic material, which can cause imaging artifacts [6,26,74]. This is particularly relevant for IE involving pulmonary or tricuspid valve prostheses, pulmonary conduits, aorto-pulmonary shunts, and cardiac implantable electronic devices (CIEDs) [3,94]. This limitation is significant as prosthetic materials are one of the leading causes of IE worldwide [3].

## 4. Role of SPECT/CT and PET/CT in CHD Patients with IE

### 4.1. SPECT/CT

Single Photon Emission Computed Tomography (SPECT)/CT remains a vital imaging modality for diagnosing IE in complex CHD cases. It provides critical diagnostic insights in challenging anatomical regions, such as prosthetic valves and CIEDs, by utilizing radiolabeled white blood cells (WBC) like technetium-99m (99mTc-HMPAO) or indium-111 (111In-oxine) [95]. This ability to pinpoint infected areas, even in difficult-to-visualize structures, makes it indispensable for CHD patients with surgical interventions involving implants or shunts [96].

A unique strength of SPECT/CT is its capability to distinguish between postoperative inflammation, which is common after multiple cardiac surgeries, and active infection. This is particularly relevant in CHD patients, where frequent surgeries to correct anatomical defects result in chronic inflammatory responses. SPECT/CT’s ability to delineate these conditions improves diagnostic precision and reduces the risk of mismanagement. Additionally, SPECT/CT excels in detecting septic emboli, even in extracardiac locations, enhancing the sensitivity of IE diagnoses. This allows for a comprehensive assessment of infection extent, influencing both diagnostic certainty and therapeutic decision-making [96].

### 4.2. PET/CT

18F-FDG Positron Emission Tomography (PET)/CT is emerging as a superior alternative to WBC SPECT/CT for evaluating IE in CHD patients due to its high photon detection efficiency, spatial resolution, and shorter imaging protocols. These advantages translate into fewer false negatives and quicker diagnostic workflows. PET/CT also plays a pivotal role in assessing disease severity and guiding therapy, making it increasingly indispensable in clinical practice [97].

**Figure 7:**
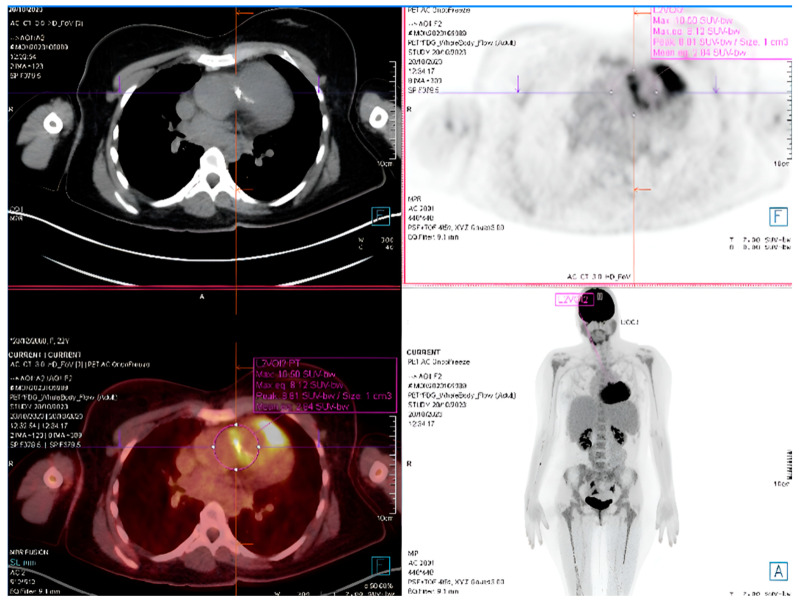
PET/CT of a patient with tetralogy of Fallot and infective endocarditis of the pulmonary conduit.

In CHD patients, PET/CT addresses significant diagnostic challenges posed by their unique and complex anatomical structures. Metal-related artifacts from prosthetic materials often limit the effectiveness of echocardiography, MRI, and even SPECT/CT. PET/CT not only overcomes these limitations but also provides comprehensive information on both intracardiac and extracardiac infection. Specifically, it excels in:

- Detecting Device-Related Infections: PET/CT is highly effective for evaluating infections involving prosthetic valves, pacemaker leads, and other intracardiac devices (Figure 7). It offers unmatched sensitivity in visualizing device pockets and lead tracks, helping differentiate sterile thrombi from infectious vegetations. This is critical in CHD patients, who frequently require surgical implants as part of their treatment.

- Enhancing Diagnostic Accuracy with the Duke Criteria: PET/CT significantly improves the sensitivity of the Duke Criteria for IE diagnoses, increasing it from 52 to 70% to 87–97%. By combining PET/CT findings with clinical and microbiological evidence, cases previously classified as “possible IE” can often be reclassified as either “definite” or “rejected”, reducing diagnostic ambiguity [27,98].
- Integration with CT Angiography (PET/CTA): When PET is paired with CT angiography, the resulting hybrid imaging achieves remarkable diagnostic precision. This combination leverages PET’s sensitivity for detecting metabolic activity in infected tissues and CTA’s detailed visualization of structural abnormalities. For instance, PET/CTA achieves a sensitivity of 91% and a positive predictive value of 93% for diagnosing infections involving prosthetic valves and intracardiac devices, significantly enhancing diagnostic confidence [99].

### 4.3. Detection of Extra-Cardiac Pathologies

PET/CT is particularly valuable in identifying extracardiac complications of IE, such as septic emboli or metastatic infections in distant organs. In CHD patients, these complications are often silent but can lead to severe clinical outcomes if undetected. PET/CT demonstrates superior sensitivity (92%) compared to SPECT/CT (75%) in detecting such embolic events [100]. Its ability to localize septic emboli in organs like the spleen, brain, and kidneys is critical for tailoring antibiotic regimens or planning surgical interventions. Moreover, PET/CT aids in identifying additional sources of infection or concurrent infections, further optimizing patient management.

### 4.4. Therapeutic Impact

The integration of PET/CT findings into clinical workflows has a profound impact on therapeutic decision-making, altering treatment strategies in over 30% of cases. This includes identifying cases requiring urgent surgical intervention or confirming the absence of active infection, thus avoiding unnecessary device extractions [101]. For instance:

- Avoiding Unnecessary Procedures: PET/CT’s ability to distinguish active infections from sterile inflammation helps prevent unwarranted removal of prosthetic devices or unnecessary surgical interventions.
- Guiding Surgical Interventions: In cases where PET/CT identifies active infections such as abscesses or large vegetations with embolic potential, surgical interventions can be prioritized to mitigate risks.
- Monitoring Treatment Response: PET/CT is also invaluable in evaluating the efficacy of antibiotic therapy by tracking changes in metabolic activity at infected sites. This early assessment allows for timely adjustments to therapeutic regimens, optimizing patient outcomes.

In conclusion, both SPECT/CT and PET/CT offer critical diagnostic and therapeutic benefits for managing IE in CHD patients. While SPECT/CT provides excellent specificity and is well-suited for distinguishing between postoperative inflammation and infection, PET/CT excels in sensitivity, speed, and the evaluation of complex cases involving prosthetic devices. Together, these imaging modalities significantly improve diagnostic accuracy, guide therapeutic decisions, and enhance outcomes for this challenging patient population. As technology advances, hybrid approaches like PET/CTA and the development of bacteria-specific radiopharmaceuticals promise to further elevate the role of nuclear imaging in managing IE in CHD patients.

## 5. Gaps in Evidence and New Perspectives

Even with great advancements, there are still significant areas that lack sufficient investigation.

- Standardization of imaging protocols

Clear guidelines on the appropriate use of different modalities in this complex population are still evolving. Optimization of imaging strategies with clear identification of which technique for which patient may overcome diagnostic challenges and allow IE identification in difficult contexts, such as the vegetation in RVOT or prosthetic valves.

- Assessment fluid dynamics

Evaluation of blood flow, fluid dynamics, and valve regurgitation is crucial for estimating disease severity and formulating therapy options. The study of blood flow fluid dynamics by echocardiographic speckle-tracking images [102], along with the use of emerging technologies such as 4D flow MRI, may provide interesting insights into the estimation of altered fluid dynamics and aid in diagnostic decision-making.

- Role of hybrid technologies

Further investigation may be warranted to evaluate the role of novel hybrid technologies, such as PET/MR imaging, in the detection of subtle inflammatory activity and the monitoring of infectious endocarditis.

- Risk models

The prognosis of this variegate population is often difficult to estimate. By combining imaging data, microbiological data, and clinical variables, specific risk stratification models could be used to predict the likelihood of complications, including embolic events, heart failure, and mortality, and help create personalized treatment strategies, ultimately improving patient outcomes.

- Telemedicine

Finally, the use of telemedicine could address disparities in access to advanced imaging technologies, particularly in resource-limited settings, to ensure equitable care for all patients with CHD [103].

## 6. Conclusions

The management of patients with IE and CHD presents unique challenges, given the complex anatomical substrates, frequent presence of prosthetic material, and potential for complications.

**Figure 8:**
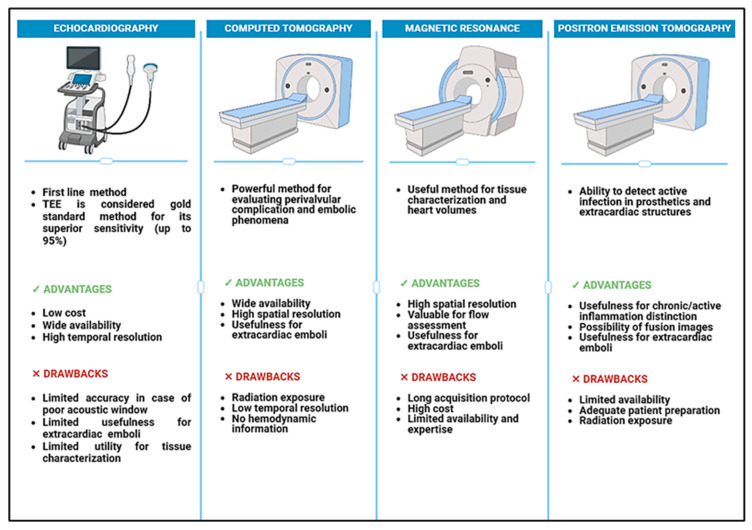
Advantages and drawbacks of different imaging modalities in patients with congenital heart disease and infective endocarditis. TEE, transesophageal echocardiography.

**Figure 9:**
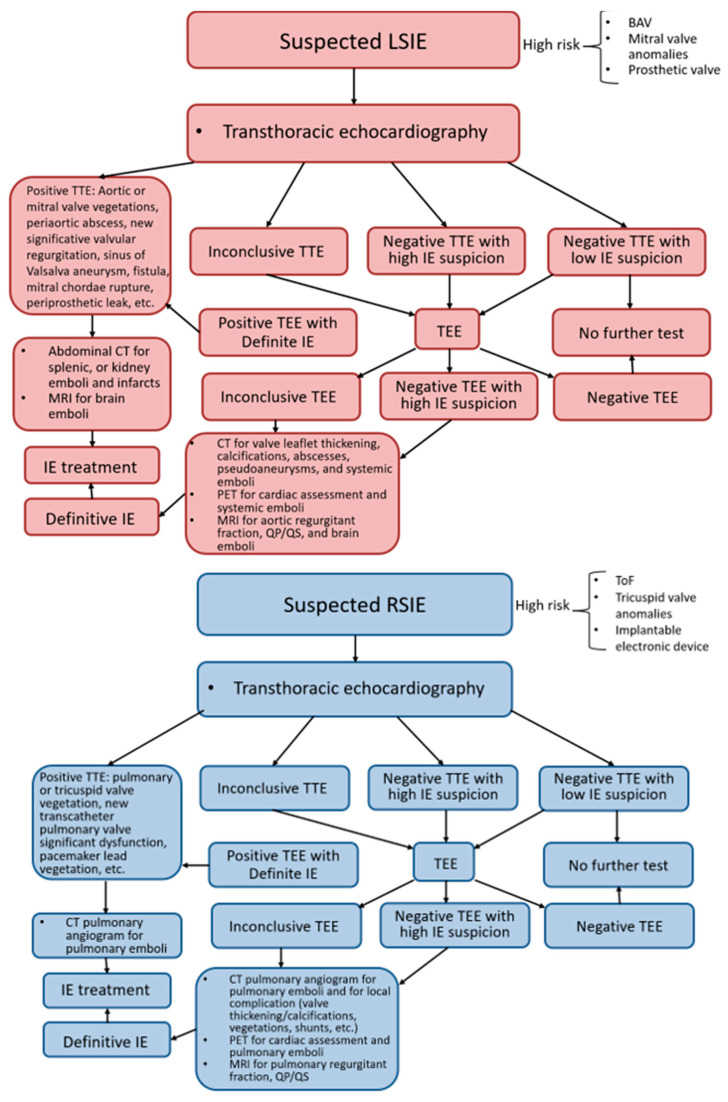
Flowchart for multi-modality imaging assessment in LSIE and RSIE. LSIE, left-side infective endocarditis; BAV, bicuspid aortic valve; TTE, trans-thoracic echocardiography; IE, infective endocarditis; CCT, cardiac computed tomography; ECG, electrocardiogram; TEE, trans-esophageal echocardiography; MRI, magnetic resonance imaging; PET, positron emission tomography; RSIE, right-side infective endocarditis; ToF, tetralogy of Fallot.

A multimodality imaging approach is essential for accurate diagnosis and optimal patient care. While echocardiography remains the cornerstone technique, CCT offers valuable insight into perivalvular complications and embolic phenomena, CMR provides valuable information on cardiac function and tissue characterization, and SPECT/CT and PET/CT allow for the detection of active infection in prosthetic material and identification of extracardiac complications (Figure 8 and Figure 9).

Integrating these imaging modalities may improve diagnostic accuracy, guide treatment decisions, and improve patient outcomes in this challenging patient population.

## Supplementary Materials

The following supporting information can be downloaded at: https://www.mdpi.com/article/10.3390/jcm14061862/s1, Supplementary S1: Video images of 2D (A,B,D,E) and 3D (C,F) transthoracic echocardiography of vegetations on aortic and mitral valve of patient with coarctation of aorta and bicuspid aortic valve.

## Abbreviations

The following abbreviations are used in this manuscript:

CHD	Congenital heart disease
IE	Infective endocarditis
CCT	Cardiac Computed Tomography
CMR	Cardiac Magnetic Resonance
BAV	Bicuspid aortic valve
LSIE	Left-sided infective endocarditis
MVP	Mitral valve prolapse
MV	Mitral valve
TEE	Transesophageal echocardiography
3D	Three-dimensional
2D	Two-dimensional
TTE	Transthoracic echocardiography
RSIE	Right-sided infective endocarditis
TV	Tricuspid valve
ToF	Tetralogy of Fallot
PV	Pulmonary valve
RVOT	Right ventricular outflow tract
4D	Four-dimensional
